# Supplementary material for: Gasdermin D cysteine residues synergistically control its palmitoylation-mediated membrane targeting and assembly
Source: EMBO J. 2024 Aug 14;43(19):4274–97. doi: 10.1038/s44318-024-00190-6 (PMC11445239; doi:10.1038/s44318-024-00190-6)
Supplement: Supplementary file 1 — Appendix [file 44318_2024_190_MOESM1_ESM.pdf]

Appendix

Gasdermin D cysteine residues synergistically control its palmitoylation-mediated membrane targeting and assembly

Eleonora Margheritis[1], Shirin Kappelhoff[1], John Danial[2,3,4], Nadine Gehle[1], Wladislaw Kohl[1], Rainer Kurre[1] , Ayelén González Montoro[1] and Katia Cosentino[1,*]

[1] Department of Biology/Chemistry and Center for Cellular Nanoanalytics (CellNanOs), University of Osnabrück, Germany

[2] Yusuf Hamied Department of Chemistry, University of Cambridge, Cambridge, United Kingdom

[3] UK Dementia Research Institute, University of Cambridge, Cambridge, United Kingdom

[4] New address: School of Physics and Astronomy, University of St Andrews, North Haugh, St Andrews, United Kingdom

* *Correspondence to: katia.cosentino@uni-osnabrück.de*

Table of Contents **Page**

Appendix Figure S1	2, 3
Appendix Figure S2	4
Appendix Figure S3	5
Appendix Figure S4	6, 7
Appendix Figure S5	8, 9
Appendix Figure S6	10, 11
Appendix Table S1	12, 13

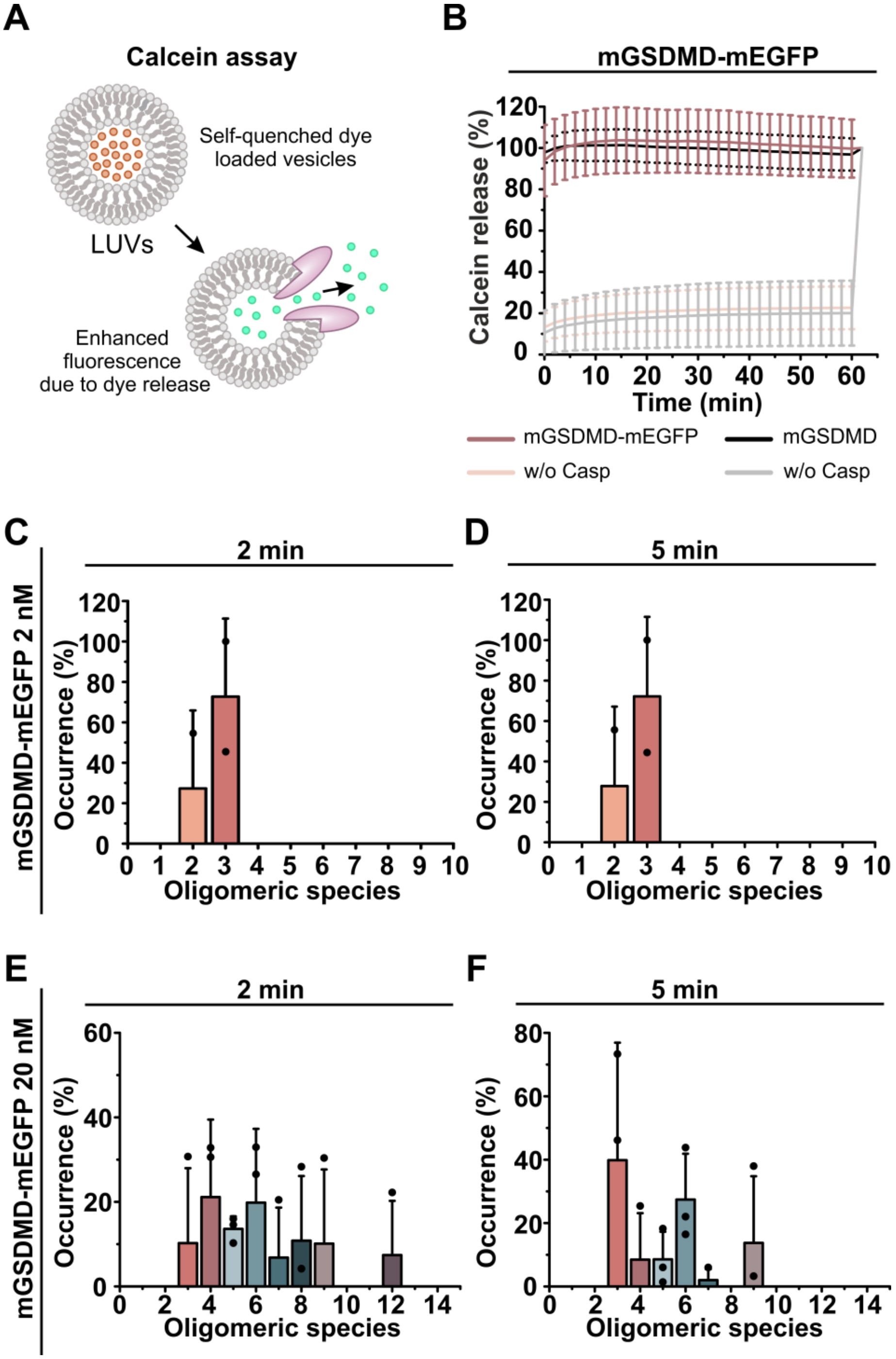

Appendix Figure S1: Different proteoliposome incubation times do not affect the process of oligomerization.

A) Schematic representation of the liposome leakage assay. mGSDMD(NTD)-mEGFP oligomers (pink) are incubated with calcein-loaded LUVs (Egg PC:PI(4, 5)P_2 99:1 mol %) and monitored over time for the ability to permeabilize the vesicles allowing the release of the fluorescent dye.

B) Liposome leakage assay as a percentage of calcein release for 60 min incubation comparing the GFP labelled protein mGSDMD-mEGFP to mGSDMD, with or without (w/o Casp) 10 nM Caspase 11. Averages from three independent experiments.
C-F) Percentage of occurrence of GSDMD oligomeric species from samples prepared from proteoliposomes incubated with low (C-D) and high concentrations (E-F) of GSDMD at different incubation time points: 2 min (C and E), 5 min (D and F) calculated as the average value from two experiments (minimum 3000 particles per experiment). Individual experimental data points are indicated as scatter plots in the graphs (0 values are not indicated).
Error bars correspond to the SD from the different experiments.

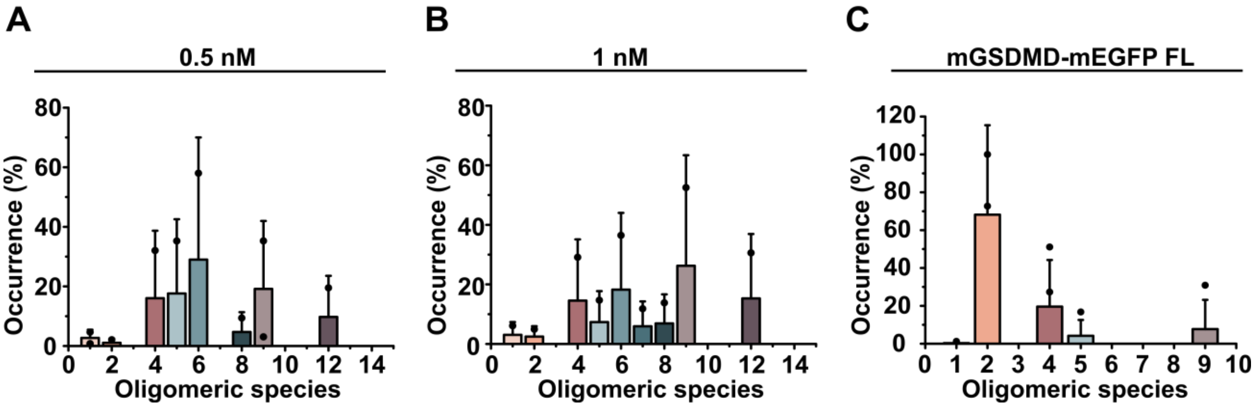

Appendix Figure S2: Oligomeric state of membrane-inserted mGSDMD-mEGFP at low concentrations and full-length protein.

A and B) Percentage of occurrence of GSDMD oligomeric species from samples prepared by incubating preformed SLBs with 0.5 nM (A) or 1 nM (B) of active mGSDMD-mEGFP. Averages from two independent experiments with a minimum of 2000 particles per experiment.

C) Percentage of occurrence of GSDMD oligomeric species from samples prepared by incubating preformed SLBs with 2 nM mGSDMD-mEGFP FL. Averages from four experiments with a minimum of 150 particles analyzed per experiment.

Individual experimental data points are indicated as scatter plots in the graphs (0 values are not indicated).

Error bars correspond to the SD from the different experiments.

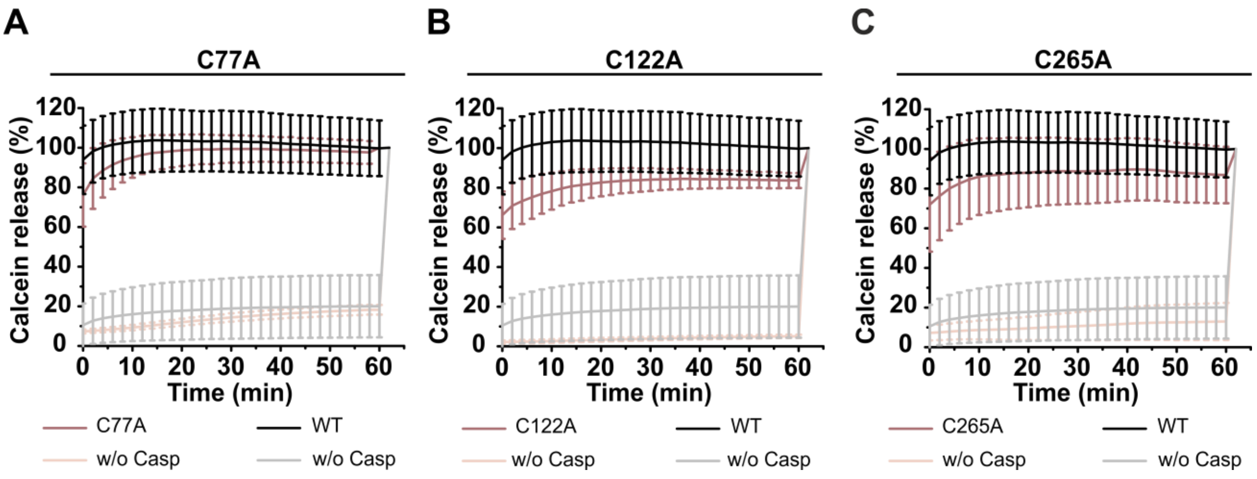

Appendix Figure S3: Calcein assays of single cysteine mutants C77A, C122A and C265A. A-C) Plots of liposome leakage assay as a percentage of calcein release for 60 min incubation with the cysteine mutants C77A (A), C122A (B) C265A (C) with or without (w/o Casp) 10 nM Caspase 11 compared to the WT mGSDMD-mEGFP (WT). Averages from at least two independent experiments and four replicates.
Error bars correspond to the SD from the different experiments.

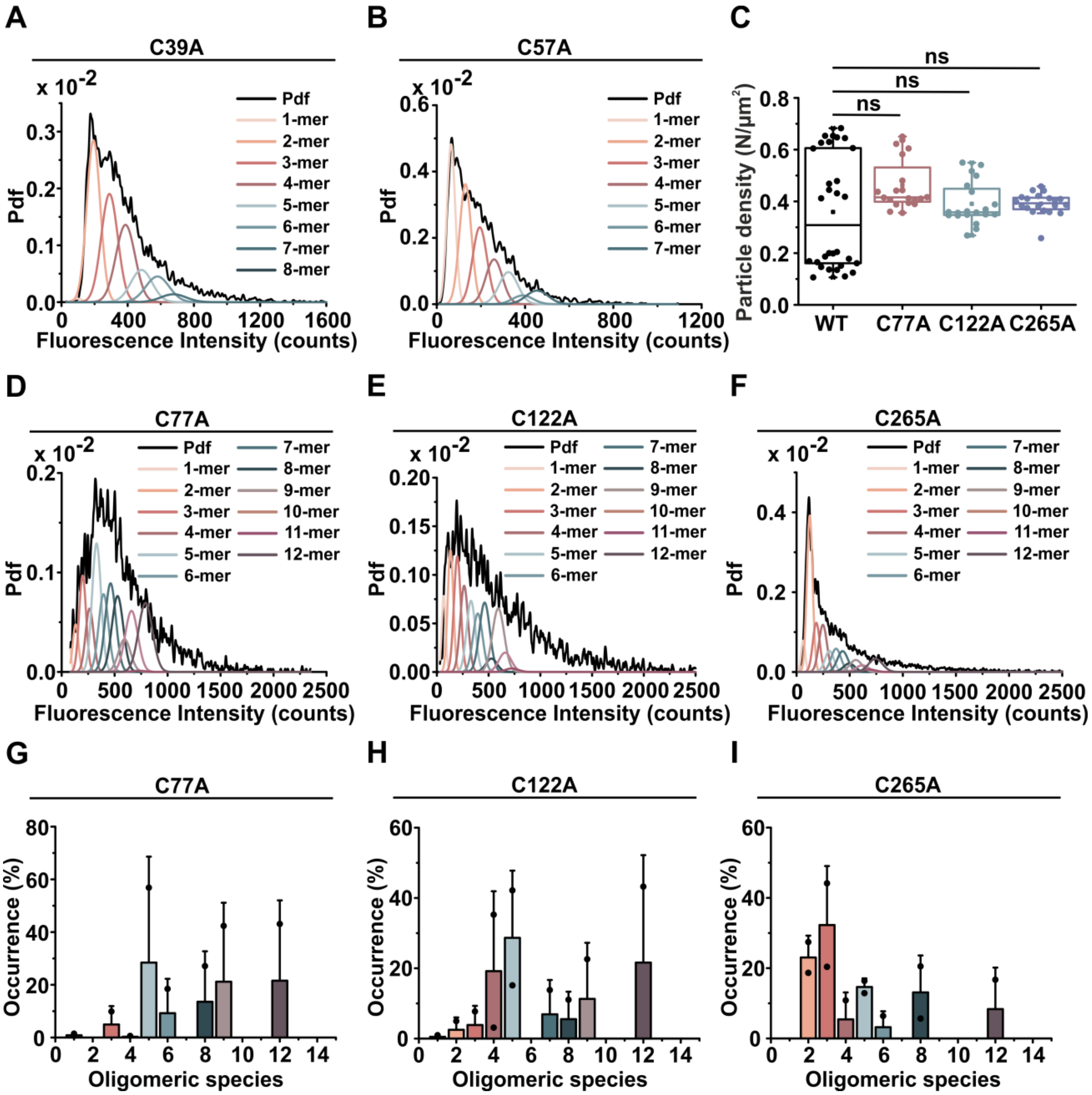

Appendix Figure S4: Stoichiometry analysis of cysteine single mutants.

A, B) Representative fluorescence distribution of oligomers of mGSDMD-mEGFP cysteine mutants C39A (A), and C57A (B) obtained from preformed SLB incubated with 2 nM of the corresponding protein activated with 20 nM Caspase 11. The resulting brightness distribution was plotted as a probability distribution function (Pdf, black) and fitted with a mixture of Gaussians to estimate the percentage of occurrence of particles containing n-mer oligomers (color).

C) Density of detected particles comparing active mGSDMD-mEGFP with the cysteine mutants C77A, C122A and C265A (C77A: p=0.052; C122A: p=0.542; C265A: p=0.523). Plotted are data from at least two different experiments.

D-F) Representative fluorescence distribution of oligomers of mGSDMD-mEGFP cysteine mutants C77A (D), C122A (E), and C265A (F) obtained from preformed SLB incubated with 2 nM

of the corresponding protein activated with 20 nM Caspase 11. The resulting brightness distribution was plotted as a probability distribution function (Pdf, black) and fitted with a mixture of Gaussians to estimate the percentage of occurrence of particles containing n-mer oligomers (color).

G-I) Percentage of occurrence of mGSDMD-C77A-mEGFP (G), mGSDMD-C122A-mEGFP (H), and mGSDMD-C265A-mEGFP (I) oligomeric species calculated as the average value from at least two experiments with a minimum of 3000 particles per experiment (the color code used for the occurrence graphs is the same as for the distributions in D-F). Data are corrected for GFP partial labeling.

Individual experimental data points are indicated as scatter plots in the graphs (0 values are not indicated).

Error bars represent SD from the different experiments. Statistics were measured by Student´s t-tests with ns (non-significant) for $p>0.05$.

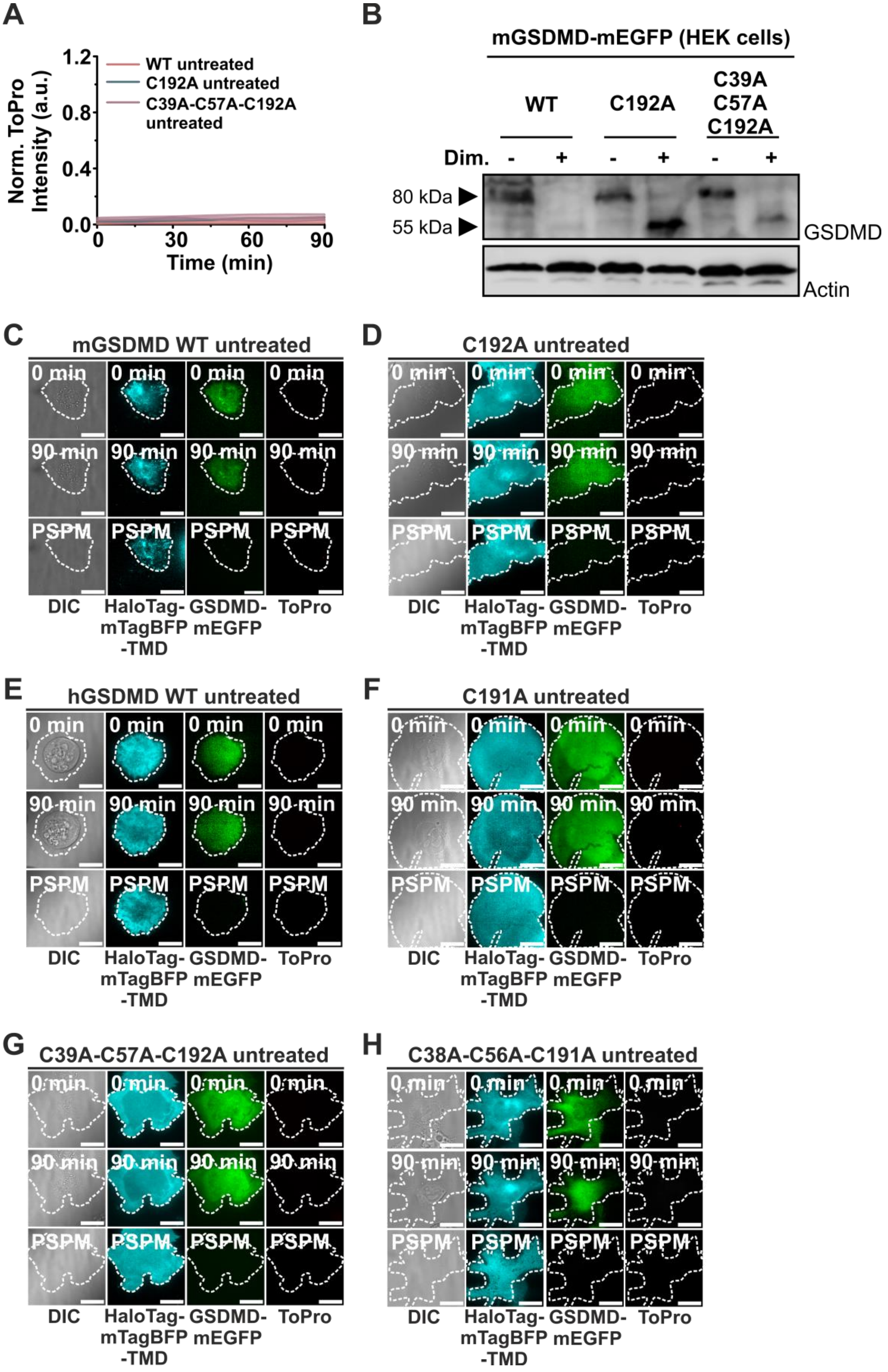
A
Norm. ToPro Intensity (a.u.)
Time (min)
WT untreated
C192A untreated
C39A-C57A-C192A untreated
B
mGSDMD-mEGFP (HEK cells)
WT
C192A
C39A C57A C192A
Dim.
80 kDa
55 kDa
GSDMD
Actin
C
mGSDMD WT untreated
D
C192A untreated
E
hGSDMD WT untreated
F
C191A untreated
G
C39A-C57A-C192A untreated
H
C38A-C56A-C191A untreated
0 min
90 min
PSPM
DIC
HaloTag-mTagBFP-TMD
GSDMD-mEGFP
ToPro

Appendix Figure S5: Cell membrane targeting controls.

A) Quantification of PM permeabilization of untreated HEK293T cells as shown in **Figure 5A** by normalized fluorescence intensity of ToPro3-Iodide (ToPro) over 90 min (*n* = 2-3 experiments with > 25 cells analyzed per experiment). Lines in the graph correspond to the average values from all measured cells and colored areas to data variability (mean ± SD).

B) Immunoblot of lysates from untreated and pyroptotic cells expressing mGSDMD-mEGFP WT, mGSDMD-C192A-mEGFP, and mGSDMD-C39A-C57A-C192A-mEGFP. β-Actin was used as loading control. Only regions of the blot with bands of interest are shown for clarity.

C-H) Representative TIRF images of untreated HEK293T cells stably transfected with mCaspase 1 and transfected with mGSDMD-mEGFP WT (C), mGSDMD-C192A-mEGFP (D), mGSDMD-C39A-C57A-C192A-mEGFP (E), hGSDMD-mEGFP WT (F), hGSDMD-C191A-mEGFP (G) and hGSDMD-C38A-C56A-C191A-mEGFP (H) (green), and HaloTag-mTagBFP-TMD (cyan) for stable tethering on a PLL-PEG-HTL-coated surface. Images without pyroptosis induction indicated by ToPro3-Iodide staining (red), morphological changes (DIC), and GSDMD-mEGFP oligomers (green), and after PSPM formation. Scale bars 20 µm.

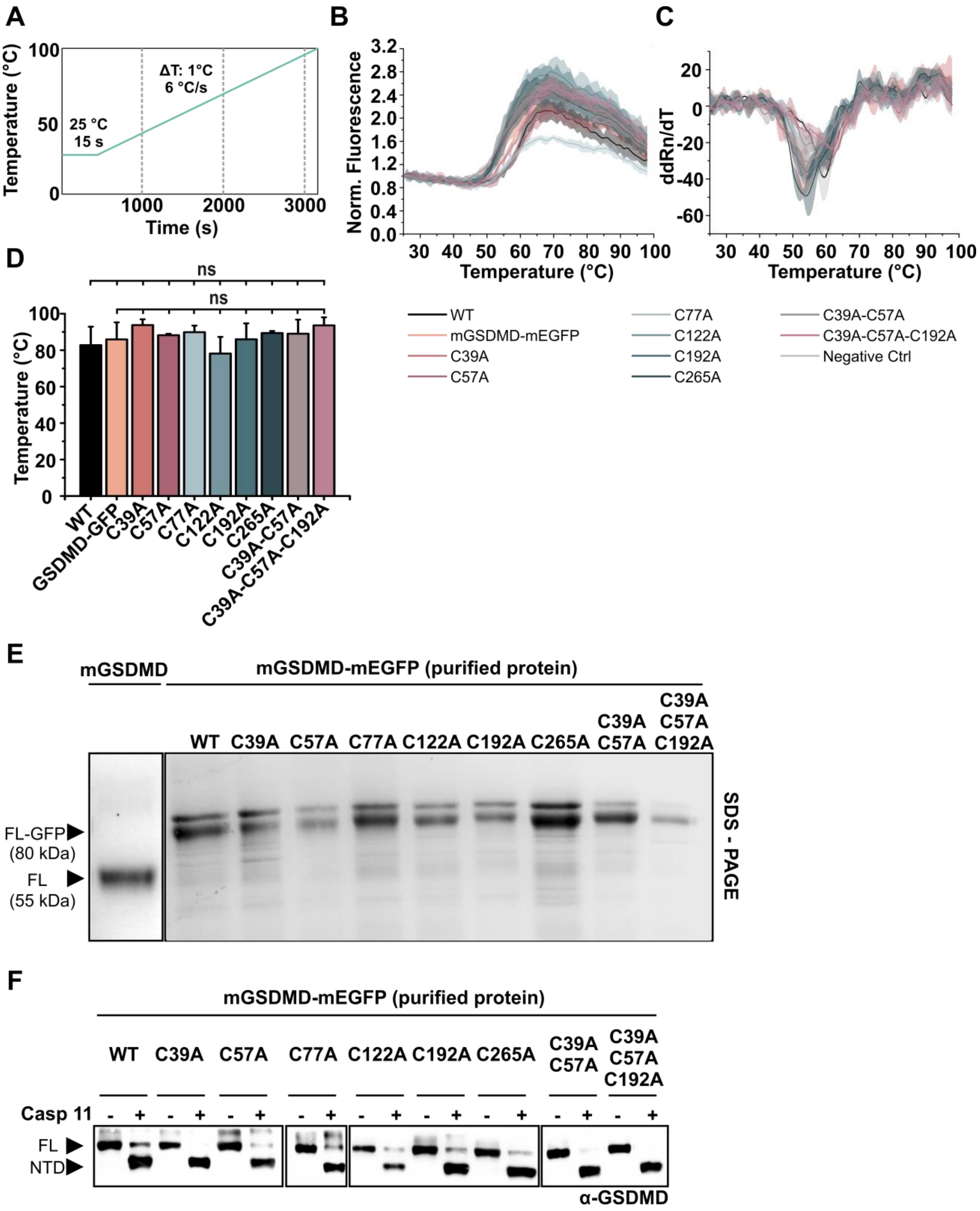

Appendix Figure S6: Purified protein quality controls.

A) Schematic representation of the protocol used for the thermal shift assay.

B-D) Thermal shift assay of mGSDMD-mEGFP WT and all the cysteine mutants. B) Thermal denaturation profile. Fluorescence intensity was normalized to the value at 25 °C. C) Plot of the

first derivative of the fluorescence emission as a function of temperature. Lines in the graph correspond to the average values from three measurements and colored areas to data variability (mean ± SD). D) Melting temperatures (T_m) identified for the different proteins from three experiments (WT/GFP labelled: *p*=0.714; WT/C39A: *p*=0.146; WT/C57A: *p*=0.406; WT/C77A: *p*=0.316; WT/C122A: *p*=0.588; WT/C192A: *p*=0.699; WT/C265A: *p*=0.321; WT/C59A-C57A: *p*=0.442; WT/C39A-C57A-C192A: *p*=0.164: GFP labelled/C39A: *p*=0.241; GFP labelled/C57A: *p*=0.693; GFP labelled/C77A: *p*=0.531; GFP labelled/C122A: *p*=0.363; GFP labelled/C192A: *p*=0.993; GFP labelled/C265A: *p*=0.556; GFP labelled/C39A-C57A: *p*=0.678; GFP labelled/C39A-C57A-C192A: *p*=0.267). Error bars correspond to the SD from the different experiments. Statistics were measured by Student´s t-tests with ns (non-significant) for *p*>0.05.
E) SDS-PAGE of the bacterial expressed proteins used in liposome leakage and stoichiometry assays.
F) Immunoblot of the bacterial expressed proteins used in liposome leakage and stoichiometry assays, before and after caspase cleavage. Only regions of the blot with bands of interest are shown for clarity.

Appendix Table S1: PCR primers

**pET21a-mGSDMD-C39A-mEGFP-8xHis**<br>**pET21a-mGSDMD-C39A-C57A-mEGFP-8xHis**<br>**pET21a-mGSDMD-C39A-C57A-C192A-mEGFP-8xHis**<br>**pSems-mGSDMD-C39A-C57A-C192A-mEGFP**	f-mGSDMD-C39A<br>GCTCTTCTGAACAGGAAATTTTCAAGCTCAAGG<br>r-mGSDMD-C39A<br>GTAGGGCCTGAAGCTGGTGGAGTTCCGCAG
**pET21a-mGSDMD-C57A-mEGFP-8xHis**<br>**pET21a-mGSDMD-C39A-C57A-mEGFP-8xHis**<br>**pET21a-mGSDMD-C39A-C57A-C192A-mEGFP-8xHis**<br>**pSems-mGSDMD-C39A-C57A-C192A-mEGFP**	f-mGSDMD-C57A<br>GTCAACCTGTCAATCAAGGACATCCTGGAGC<br>r-mGSDMD-C57A<br>AGCTGAATAACGGGGTTTCCAGAACCTTGAGC
**pET21a-mGSDMD-C77A-mEGFP-8xHis**	f-mGSDMD-C77A<br>GCCTTTGGCTCCTTCAAAGTCTCTGATGTCG<br>r-mGSDMD-C77A<br>CTCCGGTTCTGGTTCTGGAGCACTGG
**pET21a-mGSDMD-C122A-mEGFP-8xHis**	f-mGSDMD-C122A<br>GCTATACTGCGTGTGACTCAGAAGACCTG<br>r-mGSDMD-C122A<br>CACATTCATGGAGGCACTGGAACTGTC
**pET21a-mGSDMD-C192A-mEGFP-8xHis**<br>**pET21a-mGSDMD-C39A-C57A-C192A-mEGFP-8xHis**<br>**pSems-mGSDMD-C192A-mEGFP**<br>**pSems-mGSDMD-C39A-C57A-C192A-mEGFP**	f-mGSDMD-C192A<br>GGGTGAAGGCAAGGGCCACCAAAGCCGGAAG<br>r-mGSDMD-C192A<br>TTCAAGGCTAAAGCTCCAGGCAGCGTAAACTGGCC
**pET21a-mGSDMD-C265A-mEGFP-8xHis**	f-mGSDMD-C265A<br>GCCTCCATCGGAAAGCAGCTCGGAGGATC<br>r-mGSDMD-C265A<br>AAGCGCAGCAAGCACATTGAGGCCATGG
**pSems-mGSDMD-mEGFP**	f-mGSDMD-EcoRI<br>GATTAGAATTCATGCCATCGGCCTTTGAGAAAGTGGTC<br>r-mGSDMD-BamHI<br>GATTGGATCCTTATCCACAAGGTTTCTGGCCTAGACTTG
**pSems-hGSDMD-mEGFP**	f-hGSDMD-N-NheI<br>GATTAGCTAGCATGGGGTCGGCCTTTGAGC<br>r-hGSDMD-N-EcoRV<br>GATTAGATATCGTGGAGGCACCTCATCATGGAGAGG<br>f-hGSDMD-C-EcoRI<br>GATTAGAATTCAACTTCCTGACAGATGGGGTCCCTGCG

	r-hGSDMD-C-BamHI<br>GATTAGGATCCGTGGGGCTCCTGGCT<br>CAGTCCTG
**pSems-hGSDMD-C191A-mEGFP**	f-hGSDMD-C191A<br>GCCTTGCAGGGTGAGGGCCAGGGCCA<br>TC<br>r-hGSDMD-C191A<br>CGTGGCTCCGGGCAGGGAAAACCGGC
**pSems-hGSDMD-C38A-C56A-C191A-mEGFP**	f-hGSDMD-C38A<br>GCACTGGTGGTTAGGAAGCCCTCAAG<br>CTC<br>r-hGSDMD-C38A<br>GTAGGGCTGGAAGCCAGTGGAGCTCT<br>G<br>f-hGSDMD-C56A<br>GCAGTCAACCTGTCTATCAAGGACATC<br>C<br>r-hGSDMD-C56A<br>CTTATAACGGGGTTTCCAGAACCATGA<br>GC
