# Supplementary material for: Gasdermin D cysteine residues synergistically control its palmitoylation-mediated membrane targeting and assembly
Source: EMBO J. 2024 Aug 14;43(19):4274–97. doi: 10.1038/s44318-024-00190-6 (PMC11445239; doi:10.1038/s44318-024-00190-6)
Supplement: Supplementary file 3 — Movie EV1 [file 44318_2024_190_MOESM3_ESM.zip › Movie EV1/Movie EV1_legend.docx]

**Movie EV1: Tracking analysis of mobile particles of mGSDMD(NTD)-mEGFP on SLB.**

Representative video of mobile/immobile particle analysis of mGSDMD(NTD)-mEGFP on SLB analyzed by SLIMfast 4C. Mobile particles detected in the SLB sample are highlighted by white ROIs. Mobile trajectories are in color.
