# Supplementary material for: Gasdermin D cysteine residues synergistically control its palmitoylation-mediated membrane targeting and assembly
Source: EMBO J. 2024 Aug 14;43(19):4274–97. doi: 10.1038/s44318-024-00190-6 (PMC11445239; doi:10.1038/s44318-024-00190-6)
Supplement: Supplementary file 9 — Expanded View Figures [file 44318_2024_190_MOESM9_ESM.pdf]

Expanded View Figures

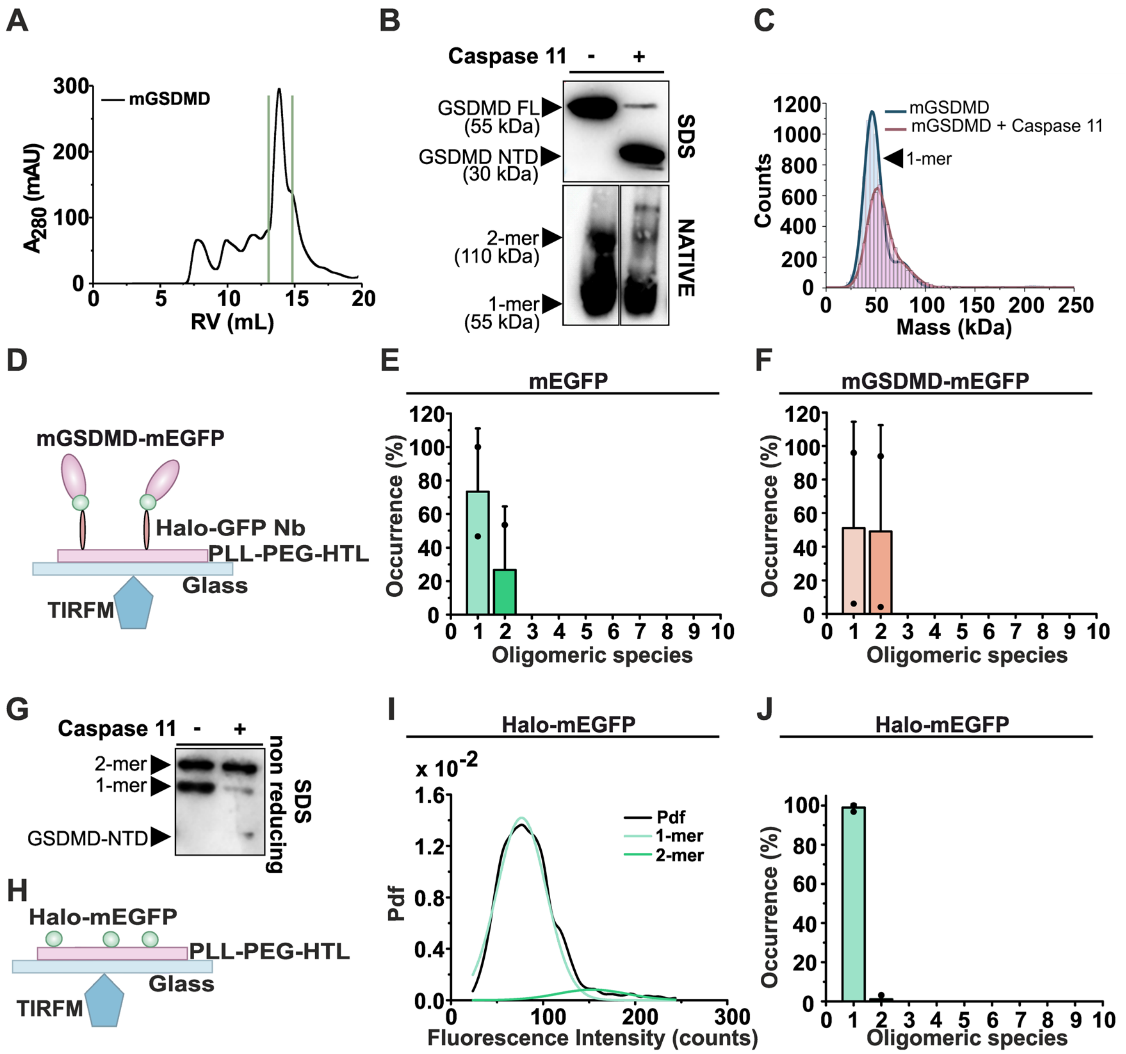

Figure EV1. Purification of mouse GSDMD and characterization of the protein in solution.

(**A**) Size-exclusion chromatography profile of recombinant full-length mouse GSDMD. (**B**) SDS and NATIVE immunoblots of full-length mouse GSDMD before and after incubation with Caspase 11. Only regions of the blot with bands of interest are shown for clarity. (**C**) Representative mass distribution of the oligomeric state of the protein before and after cleavage, analyzed by mass photometry. (**D**) Schematic representation of the sample for stoichiometry analysis of the protein in solution. The microscopy chip is functionalized with PLL-PEG-HTL (in pink) which allows the tethering of the Halo-GFP nanobody (in orange) used for the further capture of the mGSDMD-mEGFP protein (in pink and green) (see Methods). (**E**, **F**) Percentage of occurrence of mEGFP (**E**) and mGSDMD-mEGFP (**F**) oligomeric species in solution calculated as the average value from two different experiments with a minimum of 450 particles analyzed per experiment. Data are corrected for GFP partial labeling. (**G**) Immunoblot after SDS-PAGE in non-reducing conditions of full-length mouse GSDMD before and after incubation with Caspase 11. Only regions of the blot with bands of interest are shown for clarity. (**H**) Schematic representation of the system used to determine the fluorescence intensity of Halo-mEGFP used as calibration for the stoichiometric analysis. The microscopy chip is functionalized with PLL-PEG-HTL (in pink) which allows the tethering of Halo-mEGFP (green) (see Methods). (**I**) Representative fluorescence distribution of Halo-mEGFP obtained by incubating 10 pM Halo-mEGFP on a PLL-PEG-OMe: PLL-PEG-HTL 99:1 functionalized microscopy chip (see Methods). The resulting brightness distribution was plotted as a probability distribution function (Pdf, black) and fitted with a mixture of Gaussians to estimate the percentage of occurrence oligomers (color). (**J**) Percentage of occurrence of the oligomeric species of Halo-mEGFP detected confirming the monomeric state of the protein used as calibration for the stoichiometry analysis (the color code used for the occurrence graph is the same as for the distributions in (**I**)). Averages from three independent experiments with a minimum of 350 particles analyzed per experiment. Individual experimental data points are indicated as scatter plots in the graphs (0 values are not indicated). Error bars correspond to the SD from the different experiments.

 © The Author(s)

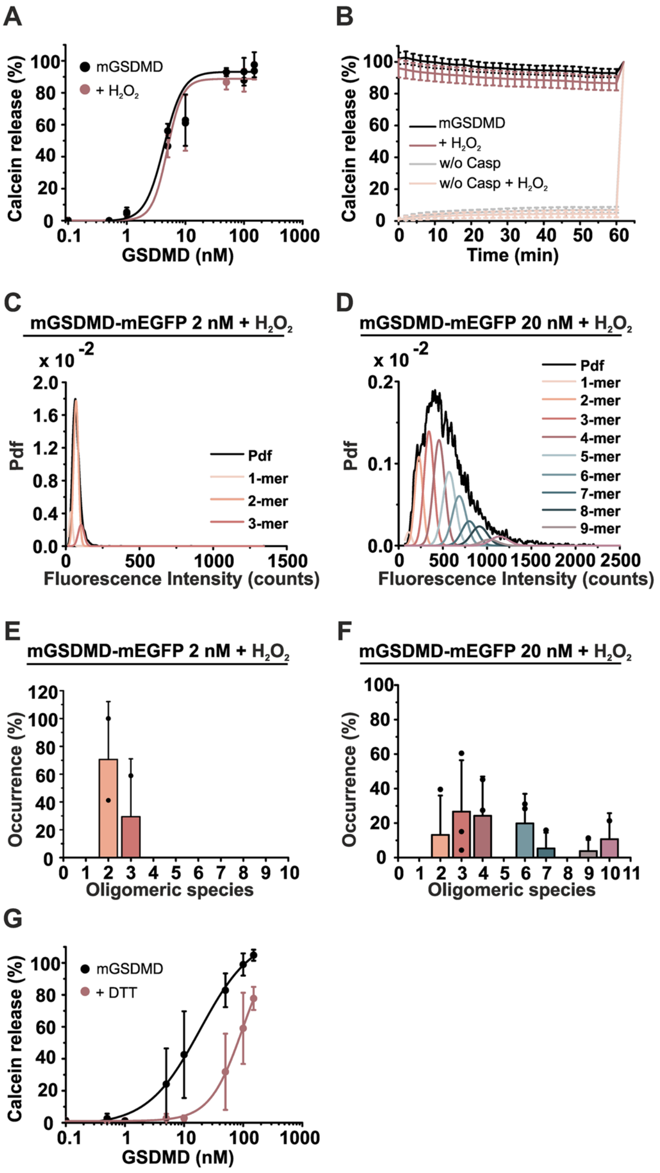

Figure EV2. Oxidation does not affect the stoichiometry of GSDMD oligomers.

(**A**) mGSDMD WT dose-dependent permeabilization of liposomes after 60 min incubation time, with or without 30 min incubation of the cleaved protein with 10 µM H_2O_2 before liposome addition. Represented are the averages from 3 independent experiments fitted with a Sigmoidal/Logistic fitting. (**B**) Liposome leakage assay as a percentage of calcein release for 60 min after incubation of liposomes with 62.5 nM mGSDMD WT with or without (w/o Casp) 10 nM Caspase 11 and pre-treated or not with 10 µM H_2O_2. Averages from three different experiments. (**C**, **D**) Representative fluorescence distribution of mGSDMD-mEGFP oligomers obtained from samples prepared with 2 nM (**C**) or 20 nM (**D**) active mGSDMD-mEGFP and incubated for 30 min with H_2O_2 before SLB formation. The resulting brightness distribution was plotted as a probability distribution function (Pdf, black) and fitted with a mixture of Gaussians to estimate the percentage of occurrence of particles containing n-mer oligomers (color). (**E**, **F**) Percentage of occurrence of the different oligomeric species of GSDMD detected (the color code used for the occurrence graph is the same as for the distributions in (**E**) and (**F**)). Averages from three independent experiments with a minimum of 2500 particles analyzed per experiment. Data are corrected for GFP partial labeling. Individual experimental data points are indicated as scatter plots in the graphs (0 values are not indicated). (**G**) mGSDMD WT dose-dependent permeabilization of liposomes after 60 min incubation time with or without 30 min incubation of the cleaved protein with 200 mM DTT before liposome addition. Averages from four independent experiments fitted with a Sigmoidal/Logistic fitting. Error bars correspond to the SD from the different experiments.

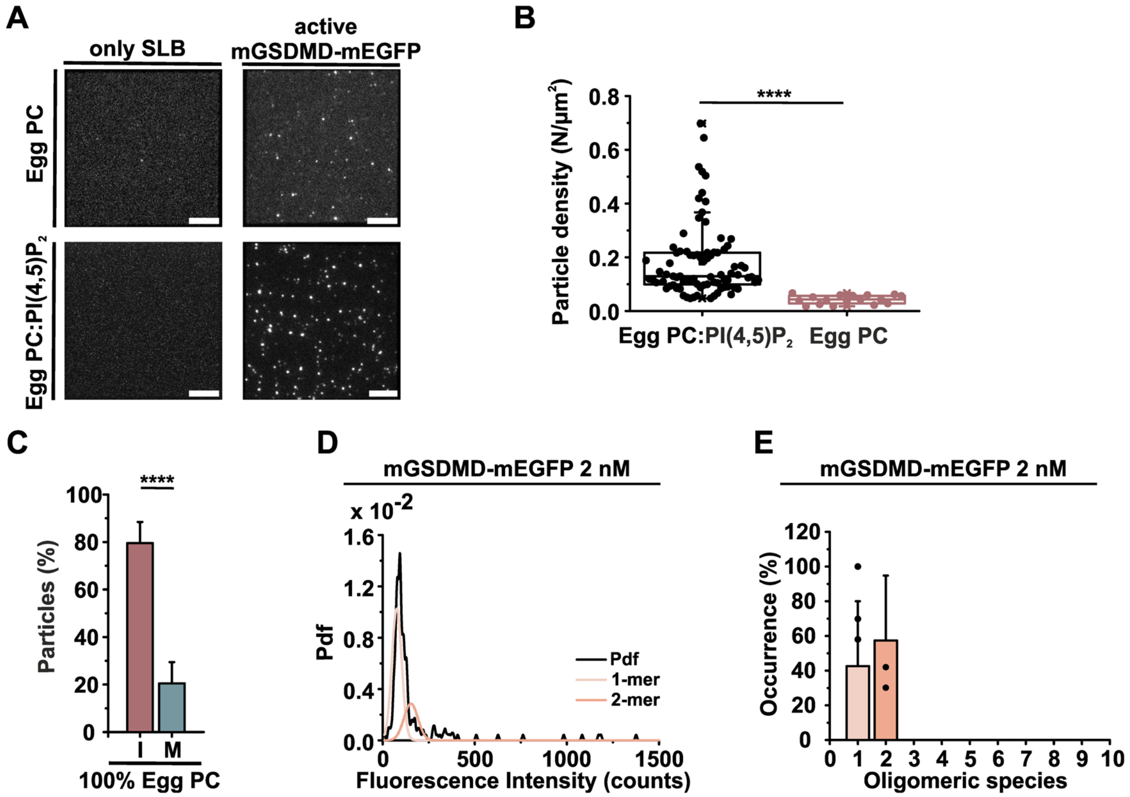

Figure EV3. Negatively charged lipids contribute to membrane binding of palmitoylated GSDMD.

(**A**) Representative TIRF images of SLB prepared with 100% Egg PC or Egg PC:PI(4, 5)P_2 99:1 mol % (left) and incubated with cleaved mGSDMD-mEGFP (right) for 1 h. Scale bars 5 μm. (**B**) Density of detected particles on SLB prepared with 100% Egg PC or Egg PC:PI(4, 5)P_2 99:1 mol %. Plotted are data from three different experiments (Egg PC:PI(4, 5)P_2 99:1: $n = 85$; 100% Egg PC: $n = 20$; $p = 3.7 \times 10^{-5}$). Data are presented as box plots with the center line at the median, lower bound at 25th percentile, upper bound at 75th percentile, and whiskers at minimum and maximum values. (**C**) Percentage of immobile (I) versus mobile (M) particles detected in the 100% Egg PC. Averages from two independent experiments with a minimum of 10 recordings analyzed per experiment ($p = 1.03 \times 10^{-13}$). (**D**) Representative fluorescence distribution of mGSDMD-mEGFP oligomers obtained from preformed SLBs prepared with 100% Egg PC and incubated with 2 nM active protein. The resulting brightness distribution was plotted as a probability distribution function (black) and fitted with a mixture of Gaussians to estimate the percentage of occurrence of particles containing n-mer oligomers. (**E**) Percentage of occurrence of the different oligomeric species of GSDMD detected. Average from two independent experiments with a minimum of 300 particles analyzed per experiment (the color code used for the occurrence graph is the same as for the distributions in (**D**)). Data are corrected for GFP partial labeling. Individual experimental data points are indicated as scatter plots in the graphs (0 values are not indicated). Error bars correspond to the SD from the different experiments. Statistics were measured by Student's t-tests with **** for $p < 0.0001$.

 © The Author(s)

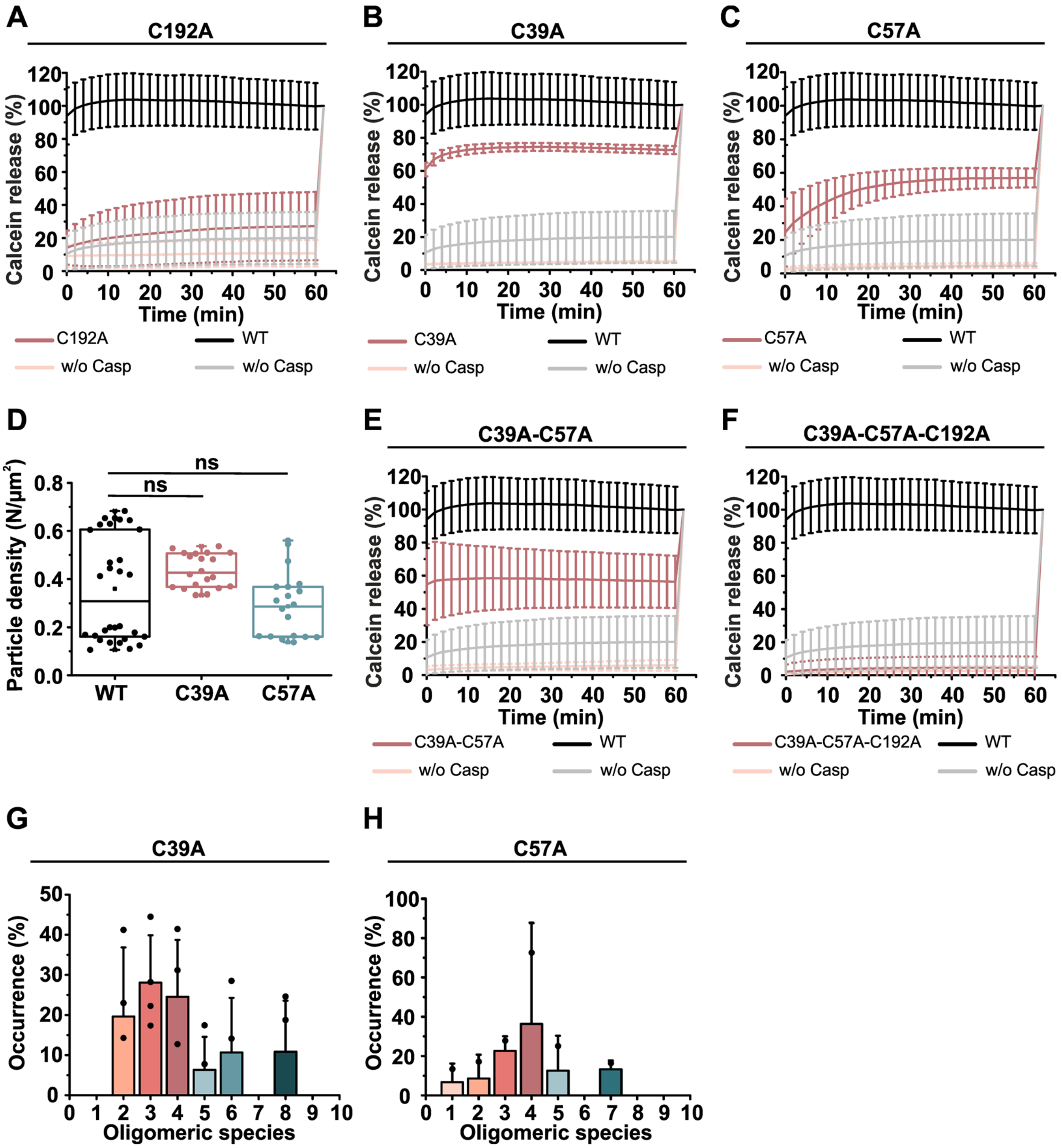

Figure EV4. Permeabilization activity and stoichiometry analysis of cysteine mutants.

(**A–C**) Liposome leakage assay as a percentage of calcein release for 60 min incubation with the cysteine mutants C192A (**A**), C39A (**B**), and C57A (**C**) with or without (w/o Casp) 10 nM Caspase 11 compared to the mGSDMD-mEGFP WT (WT) protein used as a reference for activity. Averages from at least two independent experiments. (**D**) Density of detected particles comparing active mGSDMD-mEGFP with the cysteine mutants C39A and C57A. Plotted are data from at least two different experiments (C39A: $p = 0.135$; C57A: $p = 0.196$). Data are presented as box plots with the center line at the median, lower bound at 25th percentile, upper bound at 75th percentile, and whiskers at minimum and maximum values. (**E**, **F**) Liposome leakage assay as a percentage of calcein release for 60 min incubation with the cysteine mutants C39A-C57A (**E**) and C39A-C57A-C192A (**F**) with or without (w/o Casp) 10 nM Caspase 11 compared to the mGSDMD-mEGFP WT (WT) protein used as a reference for activity. Averages from at least two independent experiments. (**G**, **H**) Percentage of occurrence of mGSDMD-C39A-mEGFP (**G**) and mGSDMD-C57A-mEGFP (**H**) oligomeric species calculated from at least two independent experiments with a minimum of 3000 particles per experiment (the color code used for the occurrence graph is the same as for the distributions in Appendix Fig. S4A,B). Data are corrected for GFP partial labeling. Individual experimental data points are indicated as scatter plots in the graphs (0 values are not indicated). Error bars correspond to the SD from the different experiments. Statistics were measured by Student's t-tests with ns (non-significant) for $p > 0.05$.

© The Author(s)

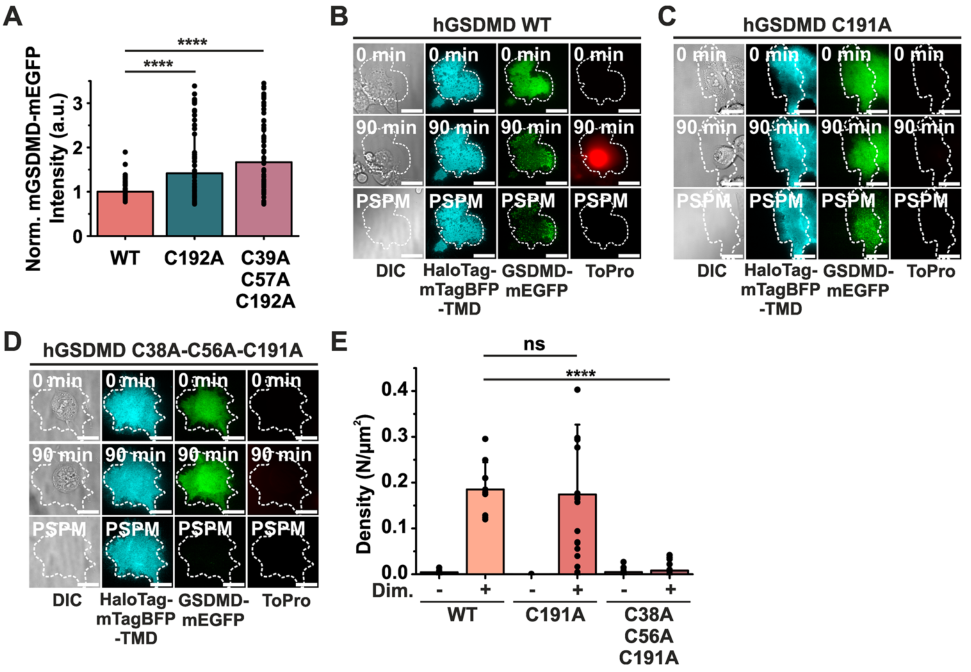

Figure EV5. Mutation at multiple cysteines abrogates GSDMD membrane targeting in cells.

(**A**) Quantification of the average expression level of stable transfected WT mGSDMD-mEGFP or transiently transfected mGSDMD-mEGFP C192A or C39A-C57A-C192A in HEK293T cells stably expressing mCaspase 1 determined by the mEGFP intensity through confocal microscopy (WT: 88 cells, 3 experiments; C192A: 93 cells, 3 experiments; C39A-C57A-C192A: 95 cells, 3 experiments) (C192A: $p = 2.13 \times 10^{-5}$; C39A-C57A-C192A: $p = 2.5 \times 10^{-10}$). Individual experimental data points are indicated as scatter plots in the graphs. (**B–D**) Representative TIRF images of HEK293T cells stably transfected with mCaspase 1 and transfected with hGSDMD-mEGFP WT (**B**), C191A (**C**) and C38A-C56A-C191A (**D**), green) and HaloTag-mTagBFP-TMD (cyan) for stable tethering on a PLL-PEG-HTL-coated surface. Images after pyroptosis induction indicated by ToPro3-Iodide staining (ToPro, red), morphological changes (DIC) and hGSDMD-mEGFP oligomers (green), and after PSPM formation. Scale bars 20 µm. (**E**) Average density of detected hGSDMD-mEGFP clusters in PSPMs (WT + Dimerizer: 9 cells, 2 experiments; WT - Dimerizer: 7 cells, 2 experiments; C191A + Dimerizer: 18 cells, 3 experiments; C191A - Dimerizer: 14 cells, 3 experiments; C38A-C156A-C191A + Dimerizer: 20 cells, 3 experiments; C38A-C56A- C191A - Dimerizer: 19 cells, 3 experiments) (C191A: $p = 0.839$; C38A-C56A-C191A: $p = 5.1 \times 10^{-13}$). Individual experimental data points are indicated as scatter plots in the graphs. Error bars represent SD from the different experiments. Statistics were measured by Student's t-tests with **** for $p < 0.0001$ and ns (non-significant) for $p > 0.05$.

 © The Author(s)
